# Retinal and Optic Nerve Integrity Following Monocular Inactivation for the Treatment of Amblyopia

**DOI:** 10.3389/fnsys.2020.00032

**Published:** 2020-06-10

**Authors:** Nadia R. DiCostanzo, Nathan A. Crowder, Braden A. Kamermans, Kevin R. Duffy

**Affiliations:** Department of Psychology & Neuroscience, Dalhousie University, Halifax, NS, Canada

**Keywords:** tetrodotoxin, retinal inactivation, ganglion cell layer, neurofilament, glial fibrillary acidic protein, myelin, glial cells, visually-evoked potentials

## Abstract

In animal models, monocular deprivation (MD) by lid closure mimics the effects of unilateral amblyopia in humans. Temporary inactivation of one or both eyes with intraocular administration of tetrodotoxin (TTX) has recently been shown to promote recovery from the anatomical effects of MD at post-critical period ages when standard recovery strategies fail. In the current study, the retinae and optic nerves of animals subjected to 10 days of monocular retinal inactivation were assessed for pathological changes as a means of assessing the viability of this potential new amblyopia therapy. Retinal sections from both eyes were subjected to hematoxylin and eosin staining and were then examined for cell density and soma size in the ganglion cell layer (GCL). Sections of the optic nerve from each eye were examined for neurofilament protein, myelin, glial cell density, and glial fibrillary acidic protein (GFAP). Our study revealed no evidence of gross histopathological abnormalities following inactivation for 10 days, nor was there evidence of degeneration of axons or loss of myelin in the optic nerve serving inactivated eyes. On all measurements, the inactivated eye was indistinguishable from the fellow eye, and both were comparable to normal controls. We confirmed that our inactivation protocol obliterated visually-evoked potentials for 10 consecutive days, but visual responses were restored to normal after the effects of inactivation wore off. Notwithstanding the critical need for further assessment of ocular and retinal health following inactivation, these results provide evidence that retinal inactivation as a treatment for amblyopia does not produce significant retinal damage or degeneration.

## Introduction

Deprivation of normal binocular vision early in postnatal life can have a profound and permanent negative consequence on the structure and function of cells in the central visual pathways (Wiesel and Hubel, 1963a,b). In cats and primates, monocular deprivation (MD) by lid closure provokes a shift in ocular dominance so that most neurons in the visual cortex become excitable exclusively by stimulation of the non-deprived eye, leaving the deprived eye able to control few neurons (Wiesel and Hubel, 1963a; Baker et al., 1974; Hubel et al., 1977). The shift in cortical ocular dominance toward the non-deprived eye originates from a weakening of deprived-eye synapses (Rittenhouse et al., 1999) that precipitates retrograde atrophy of neuron somata in the lateral geniculate nucleus (Wiesel and Hubel, 1963b) as well as shrinkage of axonal terminal fields within the visual cortex (Friedlander et al., 1982; Antonini and Stryker, 1993). The aggregation of these deprivation-induced neural abnormalities produces severe vision impairment in the deprived eye, amblyopia, which in humans is the most common cause of childhood visual impairment (Webber and Wood, 2005) that can persist even if normal visual input is restored (Dews and Wiesel, 1970; von Noorden et al., 1970; Giffin and Mitchell, 1978). The typical and long-standing treatment for amblyopia involves occluding or penalizing the stronger (fellow) eye early in postnatal life to promote recovery of the weaker (amblyopic) eye during a critical period when brain plasticity is high. In cats, monkeys, and humans, successful treatment outcomes produced by occlusion of the stronger eye decline with increasing age (Mitchell et al., 1984; Harwerth et al., 1989; Stewart et al., 2003). In humans, good recovery outcomes can be achieved when patching is implemented up until about 7 years of age, after which the efficacy of patching to promote recovery is negligible (Holmes and Levi, 2018). In cats, the effect of MD is likewise reversed only during early postnatal life. Beyond about the third postnatal month, little to no recovery is observed when the deprivation is reversed to the stronger fellow eye (Blakemore and Van Sluyters, 1974; Dürsteler et al., 1976; Movshon, 1976; Mitchell et al., 1984).

**Figure 1:**
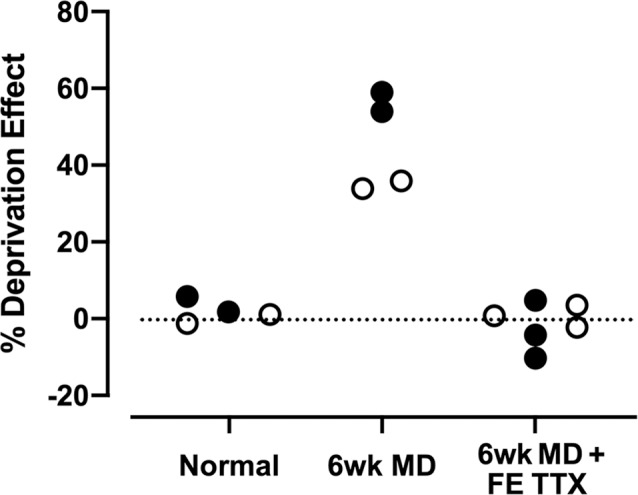
Summary of data from Duffy et al. (2018) that examined the recovery of neuron soma size (open circles) and neurofilament labeling (closed circles) in the lateral geniculate nucleus of monocularly deprived cats. Six weeks of monocular deprivation (MD) started at the peak of the critical period for plasticity produced a large deprivation effect with deprived neurons rendered about ~35% smaller than neurons connected to the non-deprived eye, and deprived layers showing a ~55% reduction in the density of neurofilament-positive neurons. Inactivation of the fellow eye for 10 days with intravitreal administration of tetrodotoxin (TTX; FE TTX) promoted a full recovery of neuron soma size and neurofilament-positive cell density in originally deprived-eye layers of the lateral geniculate nucleus.

The visual system’s age-related recalcitrance to recovery has fueled a long-standing effort to develop novel therapies for amblyopia that are effective at older ages beyond the critical period. Until recently the only manipulation that produced significant post-critical period recovery from the effects of prolonged MD in cats involved the radical step of enucleating the fellow eye. Removal of the fellow (stronger) eye elicits recovery of the weaker one and rescues behavioral, physiological and anatomical characteristics altered by amblyogenic rearing (Cragg et al., 1976; Kratz and Spear, 1976; Hoffmann and Cynader, 1977; Smith et al., 1978; Smith, 1981). Similar recovery has been documented in circumstances where the fellow eye of adult human amblyopes is removed as a result of damage or disease (Burian, 1969; von Noorden and Crawford, 1979; Rabin, 1984; Vereecken and Brabant, 1984; Klaeger-Manzanell et al., 1994). The relationship between recovery of the amblyopic eye and loss of the fellow eye was exemplified in a study of adult human amblyopes for whom the fellow eye was afflicted with age-related macular degeneration (El Mallah et al., 2000). As the disease progressed in the fellow eye and reduced its visual ability, the amblyopic eye showed a progressive recovery of visual ability, demonstrating in humans a capacity for recovery from amblyopia at older ages beyond the classically-defined critical period. Although the implications of these studies provide a basis for pursuing treatment of amblyopia at older ages, it is important to note several key limitations of the observed recovery including that characteristics of cortical receptive fields were not fully restored after enucleation, nor was visual function fully recovered following the loss of the fellow eye. These limitations notwithstanding, results of these studies demonstrate the potential for recovery at ages not responsive to typical treatments. In a recent study using cats, temporary inactivation of retinal ganglion cells in the fellow eye using tetrodotoxin (TTX), a sodium channel blocker, elicited unprecedented anatomical recovery from the effects of long-term MD, and without consequence to neurons serving the inactivated eye (Figure 1; Duffy et al., 2018). Translation of this or similar form of inactivation therapy (Fong et al., 2016) for the treatment of human amblyopia relies on the procedure being safe and without detriment to the inactivated eye. As a step toward evaluating the safety of inactivation therapy, we assessed signs of gross histopathology in the retina and optic nerve of cats following 10 days of retinal inactivation.

## Materials and Methods

### Animals

Seventeen animals were reared from birth in a closed breeding colony at Dalhousie University. Some of the animals in this study were subjects in a publication demonstrating the efficacy of fellow-eye inactivation in promoting anatomical recovery in the lateral geniculate nucleus after MD (Duffy et al., 2018). The other animals are part of ongoing studies examining recovery from longer durations of MD. The rearing history of animals and results are presented in Supplementary Table S1 (for retina) and Supplementary Table S2 (for optic nerve). We examined three groups of animals for histopathology: (1) normal animals; (2) animals that were MDed then administered TTX; and (3) animals that were MDed then administered TTX followed by a wash-out period in which binocular vision was provided (*n* = 6). The four animals that were reared under normal conditions represented our controls, two of our four control animals received intraocular vehicle injections of citrate buffer that paralleled the dosage timeline and volume parameters of our experimental groups. All animals in our experimental groups received the same 10 days of inactivation treatment. In our first experimental group, seven animals were monocularly deprived at the peak of the critical period (postnatal day 30; Olson and Freeman, 1980) for between 6 weeks and 18 weeks, which for all animals was followed by opening of the deprived eye and inactivation of the fellow eye with five intraocular injections of TTX evenly spaced across 10 days. The second experimental group contained six animals in which MD was imposed from postnatal day 30 for 6 weeks and 15 weeks, followed by opening of the deprived eye and inactivation of the fellow eye for 10 days, after which they received 10–20 days of binocular vision. Visually-evoked potentials (VEPs) were measured from the visual cortex of the fourth group of animals that contained two animals subjected to MD at postnatal day 30 for 5 weeks, then at 10 weeks of age had the right eye inactivated for 10 days with intravitreal administration of TTX. These two animals are part of a continuing study and have not been assessed for histopathology. All procedures in this study were approved by the standing committee overseeing animal care and ethics at Dalhousie University, and they adhered to use guidelines detailed by the Canadian Council on Animal Care.

### Monocular Deprivation

MD was performed under general anesthesia using 3–4% isoflurane in oxygen. The upper and lower palpebral conjunctivae of the left eye were sutured closed using vicryl suture material, which was followed by closure of the left eyelids with silk suture material. The lid closure procedure lasted about 15 min after which animals were administered subcutaneous Anafen (2 mg/kg; CDMV, Canada) as a means of postoperative analgesia, and were given topical ophthalmic Alcaine (proparacaine hydrochloride; CDMV) for local anesthesia. A broad-spectrum topical antibiotic (1% Chloromycetin; CDMV) was applied to the lids as a means to mitigate infection.

### Retinal Inactivation

Upon completion of the MD duration, animals were anesthetized with 3–4% isoflurane and the eyelids were opened followed immediately by inactivation of the fellow eye with intravitreal injection of TTX (ab120055; Abcam, Eugene, OR, USA) solubilized in citrate buffer at 3 mM. Dosage was 0.5 μl/100-g body weight but irrespective of weight, injection volume never exceeded 10 μl per injection. This dosage blocks action potentials of affected cells without obstructing critical cellular functions such as fast axoplasmic transport (Ochs and Hollingsworth, 1971). Injections were administered through a small puncture made in the sclera located at the pars plana using a sterile 30 gauge needle. Using a surgical microscope, the measured volume of TTX solution was dispensed into the vitreous chamber using a sterilized Hamilton syringe (Hamilton Company, Reno, NV, USA) with a 30 gauge needle (point style 4) that was positioned through the original puncture and about 5–10 mm into the chamber angled away from the lens. The total volume of TTX was dispensed slowly, and when complete the needle was held in place for about a minute before it was retracted. Following intraocular injection, topical antibiotics (1% Chloromycetin) and anesthetic (Alcaine) were applied to the eye to prevent post-injection complications. To achieve 10 days of inactivation, animals received five injections, one every 48 h, and for each injection, the original puncture site was used to avoid having to make another hole. A single dose of TTX administered intravitreally eliminates visual responses for at least 48 h (Wong-Riley and Riley, 1983; Stryker and Harris, 1986; Linden et al., 2009; Fong et al., 2016). During the period of inactivation, we employed basic assessments of visual behavior to confirm inactivation. We verified the absence of a pupillary light reflex as well as the lack of visuomotor behaviors such as visual placing, visual startle, and the ability to track a moving laser spot. These assessments were made while vision in the non-injected eye was briefly occluded with an opaque contact lens.

### Histology

In preparation for sectioning of the optic nerve and retina, animals were euthanized with a lethal dose of sodium pentobarbital (Euthanyl: 150 mg/kg; CDMV) and then perfused transcardially with 150 ml of phosphate-buffered saline (PBS) followed by an equal volume of 4% paraformaldehyde dissolved in PBS. Immediately after perfusion, the brain and optic nerves were removed and immersed in a PBS solution containing 30% sucrose to cryoprotect tissues for sectioning. The left and right eyes from six animals (see Supplementary Table S1) were removed, placed in 4% paraformaldehyde dissolved in PBS solution, and were then shipped to StageBio (Mt Jackson, VA, USA) for paraffin embedding and pathological assessment. At StageBio, sagittal sections through the globe of the eye were cut at a thickness of 5 μm. Sections were categorized as coming from either: the (i) center of the eye (approximately through the pupil); or the (ii) nasal; or (iii) temporal calottes. Slices of the retina were then exposed to hematoxylin and eosin (H and E) stain for histopathological observation by an ocular histopathologist at StageBio, and later for stereological quantification of cell size and density within the ganglion cell layer (GCL) by the authors of this study.

From each eye examined, pieces of the optic nerve that extended about 5 mm posterior of the lamina cribrosa were collected for processing. Cross-sections of the right and left optic nerves were cut at 50 μm using a freezing microtome and were thereafter stained with cresyl violet to reveal Nissl substance that enabled demarcation of glial cell bodies. Separate sections of the optic nerve were exposed to Luxol blue staining to reveal myelin or were immunolabeled for the cytoskeletal proteins neurofilament or glial fibrillary acidic protein (GFAP). For each marker, sections of the right and left optic nerve from all animals were mounted onto glass slides and processed together to ensure consistency in the application of histology protocols. Sections destined for Nissl staining were mounted onto glass slides, then were dehydrated in a graded alcohol series before being immersed into a 1% Nissl staining solution (ab246817; Abcam). Nissl-stained sections were differentiated with 70% ethanol, cleared with Histoclear (Diamed, Canada), and coverslipped using Permount curable mounting medium (Fisher Scientific, Canada). Sections destined for Luxol staining were mounted onto glass slides before being washed in distilled water, then were immersed in luxol blue stain (ab150675; Abcam) for 2 h at 60°C. Following luxol staining, sections were rinsed thoroughly with distilled water and then differentiated with 0.05% lithium carbonate solution (ab150675; Abcam). Luxol-stained sections were dehydrated in a graded ethanol series, cleared in Histoclear solution, and then coverslipped with Permount mounting medium. Immunolabeling for neurofilament (SMI-32; BioLegend, San Diego, CA, USA) and GFAP (ab7260; Abcam) was performed on separate sections and involved incubation of the sections in the respective primary antibody solution (1:1,000 in PBS) for 12 h, followed by incubation with either a goat anti-mouse (for neurofilament) conjugated Alexa Fluor 488 (115-585-003; Jackson Immunoresearch, West Grove, PA, USA), or a goat anti-rabbit (for GFAP) conjugated Alexa Fluor 594 (111-585-003; Jackson Immunoresearch) secondary antibody. Immunolabeled sections were mounted onto glass slides and coverslipped with a curable antifade mounting medium (Prolong Gold; Thermo Fisher, Waltham, MA, USA).

### Visually-Evoked Potentials

In preparation for measurement of VEPs, animals were initially anesthetized with 3% isoflurane in oxygen, which was reduced to between 1% and 1.5% during recordings. Supplemental sedation was provided during recordings [acepromazine (0.06–0.1 mg/kg) and butorphanol (0.1–0.2 mg/kg); I.M.]. The hair on the head was trimmed and a disposable razor was used to shave parts of the scalp where recording sites were located, one positioned approximately 2–8 mm posterior and 1–4 mm lateral to interaural zero over the presumptive location of the left primary visual cortex (contralateral to the inactivated eye), and another site over the midline of the frontal lobes that acted as a reference. Electrode sites were abraded with Nuprep EEG skin preparation gel (bio-medical, Ann Arbor, MI, USA), and were then further cleaned with alcohol pads. Reusable 10 mm gold cup Grass electrodes (FS-E5GH-48; bio-medical) were secured to each electrode site using Ten20 EEG conductive paste (bio-medical, USA) that was applied to the scalp. The impedance of the recording electrode was measured in relation to the reference electrode to ensure values were below 5 kΩ. Electrophysiological signals were amplified and digitized with an Intan headstage (RHD2132; 20 kHz sampling frequency), then recorded using an Open Ephys acquisition board and GUI software (Open Ephys, Hamden, CT, USA). Visual stimuli were programmed in MatLab using the Psychophysics Toolbox extension (Brainard, 1997; Pelli, 1997), and presented on an LCD monitor (Dell 210-AMSR; 25″ display, 240 Hz refresh, 1,920 × 1,080 pixels) at a viewing distance of 50 cm. Steady-state VEPs were elicited with full contrast square-wave gratings with a 2 Hz contrast reversal frequency (Bonds, 1984; Pang and Bonds, 1991; Norcia et al., 2015). Gratings of different spatial frequencies (0.05, 0.1, 0.5, 1, and 2 cycles /degree) or a blank gray screen were presented in random order for 20 s each, with a blank gray screen also displayed during the 2 s interstimulus interval. Each stimulus was repeated for at least six repetitions. The right eye was tested in isolation by placing a black occluder in front of the left eye during recording. The viewing eye was kept open with a small speculum, and the eyes were frequently lubricated with hydrating drops. Recording sessions lasted about 1 h and animal behavior was observed for at least an additional hour post-recording to ensure a complete recovery. The raw electroencephalogram was imported to MatLab where it was high-pass filtered above 1 Hz, then subjected to Fourier analysis (Bach and Meigen, 1999; Norcia et al., 2015). The magnitude of VEPs was calculated as the sum of the power at the stimulus fundamental frequency plus six additional harmonics (2, 4, 6, 8, 10, 12, and 14 Hz). Baseline nonvisual activity was calculated as the sum of the power at frequencies just offset from the visual response (2.45, 4.45, 6.45, 8.45, 10.45, 12.45, and 14.45 Hz).

### Quantification

The soma area of cells within the GCL as well as glial cells within the optic nerve was measured using the nucleator stereology probe from a computerized stereology system (Visiopharm, Denmark) that permitted unbiased sampling of sections being measured. From the H and E stained GCL, measurement of soma cross-sectional area was taken from cells within the central retina, as well as from the nasal and temporal calottes. Using a 60× objective, only cells with a distinctly stained nucleolus were selected for measurement to ensure measurements were taken from the somal midline, the presumed maximal diameter of the cell. Following the use of the nucleator stereology probe, the nucleus of cells selected for measurement was marked as a reference point from which four half-lines projected outward. Points along these half-lines were marked where each line crossed the outermost boundary of the soma, and these limits were used to estimate cross-sectional soma area using the following formula: area = πl^2^; where l refers to the mean length of the four arrays that extend from the nucleolus to the denoted intersection with the soma membrane. From Nissl-stained sections of the optic nerve, microscopic identification of glial cells with an obvious nucleus was selected for counting to ensure that sampled cells were sliced through the somal midline and were not a cell cap. Cell counting was achieved using the optical dissector stereology probe. For each optic nerve, counts were divided by the area sampled to calculate the density of cells. All measurements in this study were taken blind to the animal condition as well as to which eye was being examined. For each animal, an ocular dominance index was calculated by subtracting the sum of the right optic nerve measurements from the sum of the left (inactivated) optic nerve measurements, then dividing the result by the sum of the left optic nerve measurements and multiplying by 100. The final product of the metric indicated the percentage difference between left and right optic nerves.

Ocular Dominance Index: [(left optic nerve − right optic nerve)/left optic nerve] × 100

Fluorescence produced by neurofilament and GFAP immunolabeling, as well as staining produced by luxol dye, was quantified according to the following procedures. Cross-sections of the optic nerve from each animal were segmented into five square regions (69,000 μm^2^ each): dorsal, ventral, medial, lateral, and central. High magnification images from the cross-sectional segments were captured using a 60× objective for quantification, producing five images for each optic nerve. For each image, the mean gray value was calculated using ImageJ software (Schneider et al., 2012) and the five gray values for each optic nerve were averaged to quantify labeling/staining intensity for that nerve. All measurements were taken blind to animal condition as well as to which eye was being examined. These values were used to calculate an ocular dominance index for each animal employing the formula described earlier.

### Statistical Analyses

For each of our dependent measures, the extent of difference between the left and right optic nerve within each experimental condition was evaluated using the ocular dominance index that indicated the extent of difference between the eyes for each animal and each marker. Statistical assessments were made using Prism (GraphPad, La Jolla, CA, USA) and were performed on the raw data from the retina and optic nerve (Supplementary Tables S1, S2) and employed a two-tailed Kruskal–Wallis nonparametric analysis of variance using Dunn’s multiple comparisons test. A Kruskal–Wallis statistic with Dunn’s multiple comparisons test was also used to evaluate differences in the ocular dominance index between our three histology groups.

## Results

### Retina

**Figure 2:**
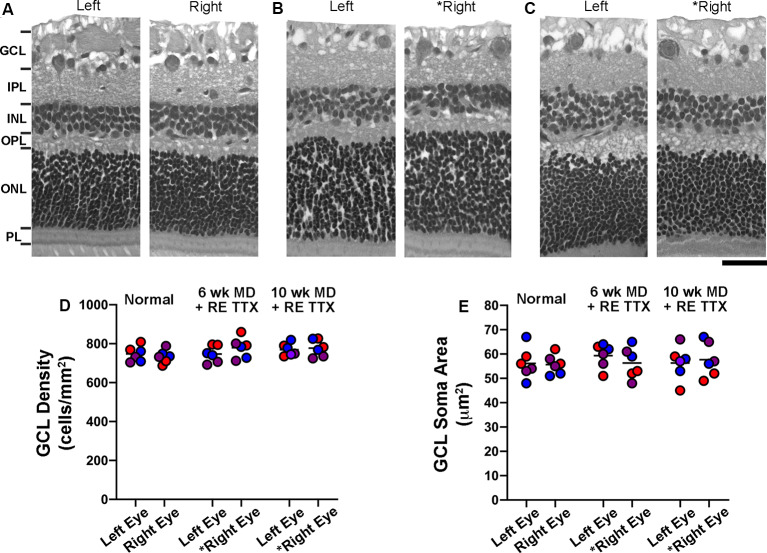
Stereological quantification of cell density and cross-sectional soma area in the ganglion cell layer (GCL) following 10 days of monocular retinal inactivation. Retinal cross-sections from the left and right eye of a normal animal **(A)** and two animals subjected to MD for either 6 **(B)** or 10 **(C)** weeks both followed by retinal inactivation for 10 days. Measurement of cell density within the GCL revealed no difference between left and right eyes of normal animals, nor was there a difference in cell density between the retinae of monocularly deprived animals that had their right eyes inactivated **(D)**. Quantification of retinal ganglion cell soma size also revealed no difference between the left and right eyes of normal animals or those subjected to monocular retinal inactivation **(E)**. Colored symbols in **(D,E)** represent measurements taken from sections of retina cut through the nasal calotte (blue), temporal calotte (purple), and central retina (red). Asterisks indicate the inactivated eye. Scale bar = 40 microns.

In addition to the quantitative histological assessments presented in this study, the left and right retinae from six animals (see Supplementary Table S1) were independently examined by a certified anatomic pathologist at StageBio (Mt. Jackson, VA, USA), a GLP-compliant preclinical histopathology provider. The pathology report from StageBio indicated that there were no microscopic abnormalities upon inspection of the retinas from untreated control eyes or those that were inactivated with TTX. Retinal sections exposed to H and E stain were obtained from StageBio for our histological assessment, which employed stereological probes to measure cell density in the GCL of each retina, as well as cross-sectional soma size from cells in the GCL. Consistent with the pathology report obtained from StageBio, our qualitative impression of the H and E processed sections was that the anatomical characteristics made visible by the stain were comparable between sections from the untreated and TTX-treated eyes, and there was no obvious difference compared to the normal control group (Figures 2A–C). Of particular interest to us was the GCL, whose cells are the principal target of the inactivation treatment. Upon microscopic inspection, the size and distribution of cells within the GCL were comparable between sections from the left and right eye in normal controls, as well as in animals whose right retina was inactivated for 10 days. To investigate whether inactivation treatment produced a change in cell density within the GCL of inactivated retinae, we stereologically counted cells made visible by H and E stain (Figure 2D), and our estimates were in line with those published previously (Spear and Hou, 1990). Cell density measurements obtained from three sagittal sections from each retina (see “Materials and Methods” section) indicated no statistical difference between left and right eye of controls or TTX-treated animals [*H* = (6) = 5.19, *p* = 0.39; Dunn’s multiple comparison test: *p* > 0.05]. Also, no statistical difference was found when density measurements from the right eye of control animals were compared to measurements from the inactivated (right) eye of TTX-treated animals (Dunn’s multiple comparison test: *p* > 0.05). We next examined the cross-sectional soma size within the GCL because this morphological alteration is an indicator of degeneration (Weber et al., 1998). Our measurements of the cross-sectional soma area were similar to those published previously from cats (Spear and Hou, 1990). In line with our cell density observations, we found no difference between the size of cells from the left and right eyes of control or TTX treatment groups [Figure 2E; *H* = (6) = 1.69, *p* = 0.88; Dunn’s multiple comparison test: *p* > 0.05]. There was also no statistical difference in soma size between the right eye of controls and the inactivated (right) eyes of either treatment group (Dunn’s multiple comparison test: *p* > 0.05).

### Optic Nerve

**Figure 3:**
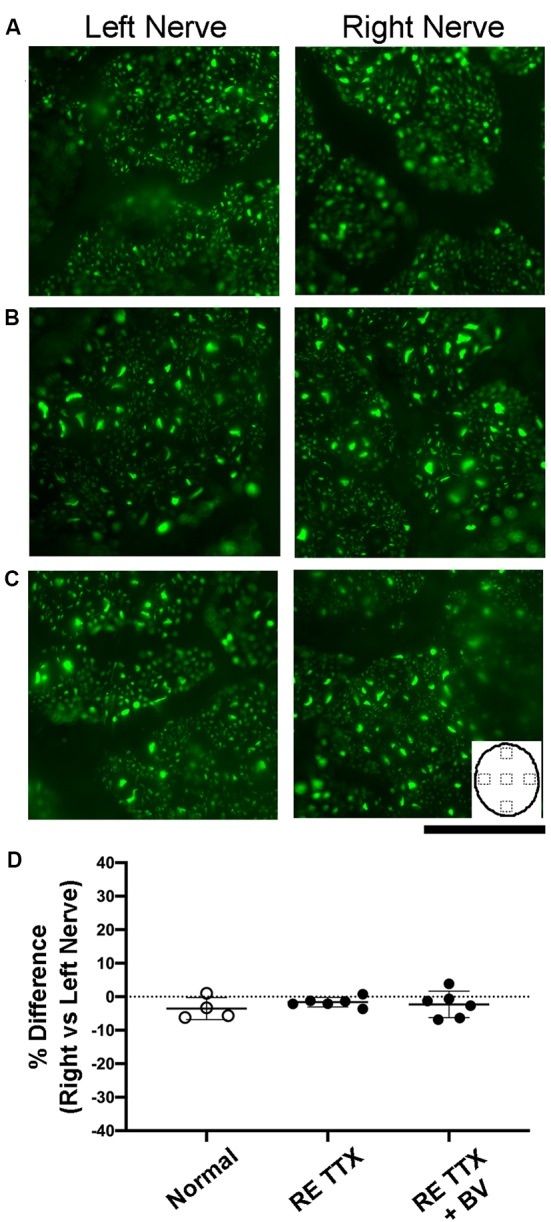
Examination of neurofilament labeling in the left and right optic nerves following inactivation of the right eye. In normal control animals, neurofilament immunofluorescence was observed to be balanced between the left and right optic nerves **(A)**. Comparable neurofilament labeling between the left and right nerves was also evident in animals subjected to inactivation of the right eye for 10 days **(B)**, as well as in animals that were likewise treated and then permitted binocular vision after inactivation wore off **(C)**. Inset in C depicts an outline of the optic nerve cross-section to illustrate the location of labeling measurements. Quantification of neurofilament fluorescence **(D)** was expressed with an ocular index that was calculated for each animal and revealed that immunolabeling from the left and right optic nerves was balanced and not statically different between groups (*H* = = 0.76, *p* = 0.70; Dunn’s multiple comparison test: *p* > 0.05). Scale bar = 250 microns.

Disruption of neurofilament labeling in the optic nerve can result from retinal degeneration provoked by experimentally-induced glaucoma (Noristani et al., 2016), and reduced neurofilament occurs during normal gestational development during which time there is a natural decline in retinal ganglion cell axons (Williams and Chalupa, 1983). Accordingly, loss of neurofilament provides a marker for axonal disruption and reduction in the optic nerve. Within our control group of animals, neurofilament labeling intensity and distribution between the left and right optic nerves were comparable and appeared balanced (Figure 3A). Animals subjected to TTX treatment in the right eye exhibited an equivalent level of neurofilament across sections from the left and right optic nerves (Figure 3B), and this was indistinguishable from the optic nerves of our control animals. In the group of animals that received binocular vision after monocular inactivation, neurofilament in the left and right optic nerves likewise appeared normal (Figure 3C), indicating there was no obvious disruption of labeling after TTX treatment wore off. In control animals, quantification of neurofilament labeling intensity across the left and right optic nerves revealed similar measurements, and this was revealed by calculation of an ocular dominance index that demonstrated balanced labeling across nerves, indicated by values close to zero (Figure 3D). Neurofilament labeling intensity was not significantly different between the left and right optic nerves across groups [*H* = (6) = 6.22, *p* = 0.28; Dunn’s multiple comparison test: *p* > 0.05], nor was there a difference between the inactivated (right) eyes of our TTX groups when compared with normals (Dunn’s multiple comparison test: *p* > 0.05).

**Figure 4:**
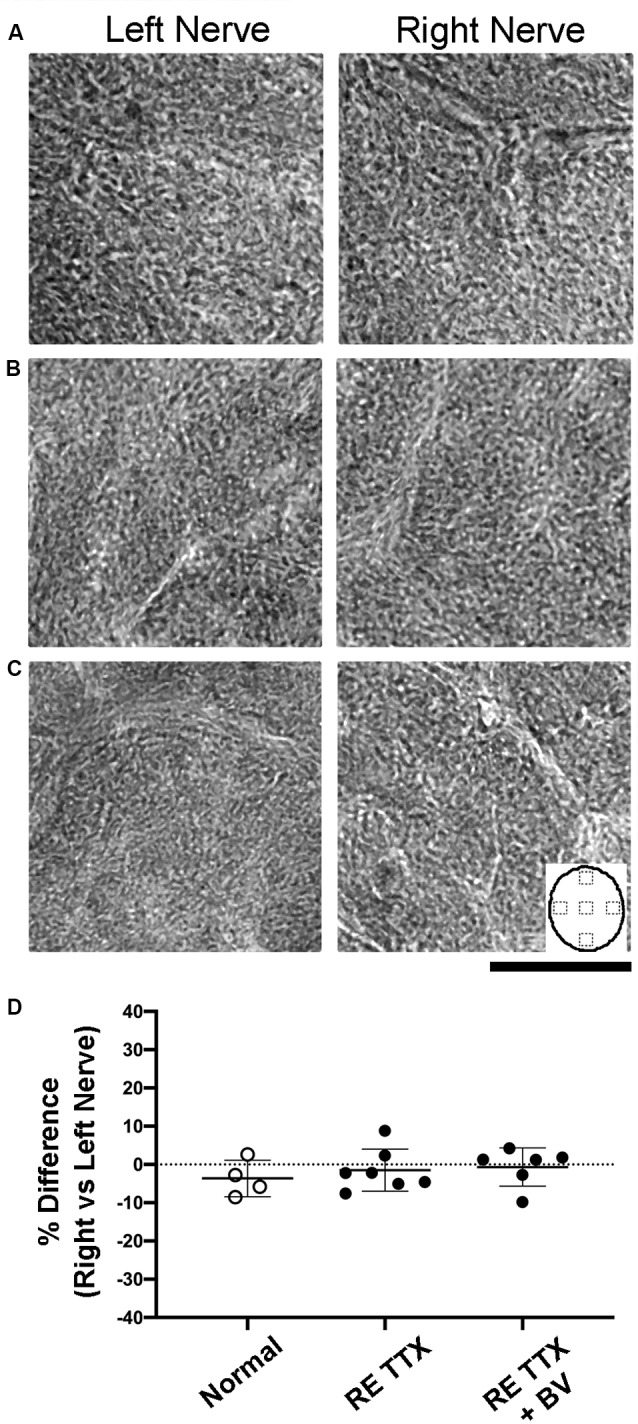
Cross-sections of the optic nerve from the left and right eye stained with luxol blue to evaluate myelin levels. In normal animals **(A)** no difference in myelin staining was observed between the left and right optic nerves. Myelin staining following right eye inactivation was likewise similar between left and right optic nerves and was comparable to normal immediately after 10 days of inactivation **(B)** or after inactivation wore off **(C)**. Quantification of myelin staining from sample locations of optic nerve cross-sections (inset in **C**) revealed a balance in staining intensity across the left and right optic nerves **(D)** that was not statistically different between groups (*H* = = 0.95, *p* = 0.64; Dunn’s multiple comparison test: *p* > 0.05). Scale bar = 250 microns.

The modification of myelin is a hallmark of optic nerve degeneration that has been demonstrated across ocular pathologies (Horstmann et al., 2013; Renner et al., 2017). We stained cross-sections of the optic nerve for myelin using luxol fast blue to assess possible myelin disruption after retinal inactivation treatment. In control animals, luxol staining was comparable between sections from the left and right optic nerves (Figure 4A). We also observed no difference in myelin staining between sections from left and right optic nerves of animals subjected to inactivation of the right eye (Figure 4B), or in animals whose right eye inactivation was followed by binocular vision (Figure 4C). Quantification of luxol staining intensity revealed similar measurements between the left and right optic nerves of control animals, which was also observed for both groups of animals that received the right eye inactivation for 10 days (Figure 4D). There was no statistical difference in luxol intensity measurements between the left and right optic nerves for the control group or either of our TTX groups [*H* = (6) = 7.70, *p* = 0.17; Dunn’s multiple comparison test: *p* > 0.05]. We also found no statistical difference in luxol staining between the right optic nerve of our control group and the treated (right) optic nerve of our TTX groups (Dunn’s multiple comparison test: *p* > 0.05).

**Figure 5:**
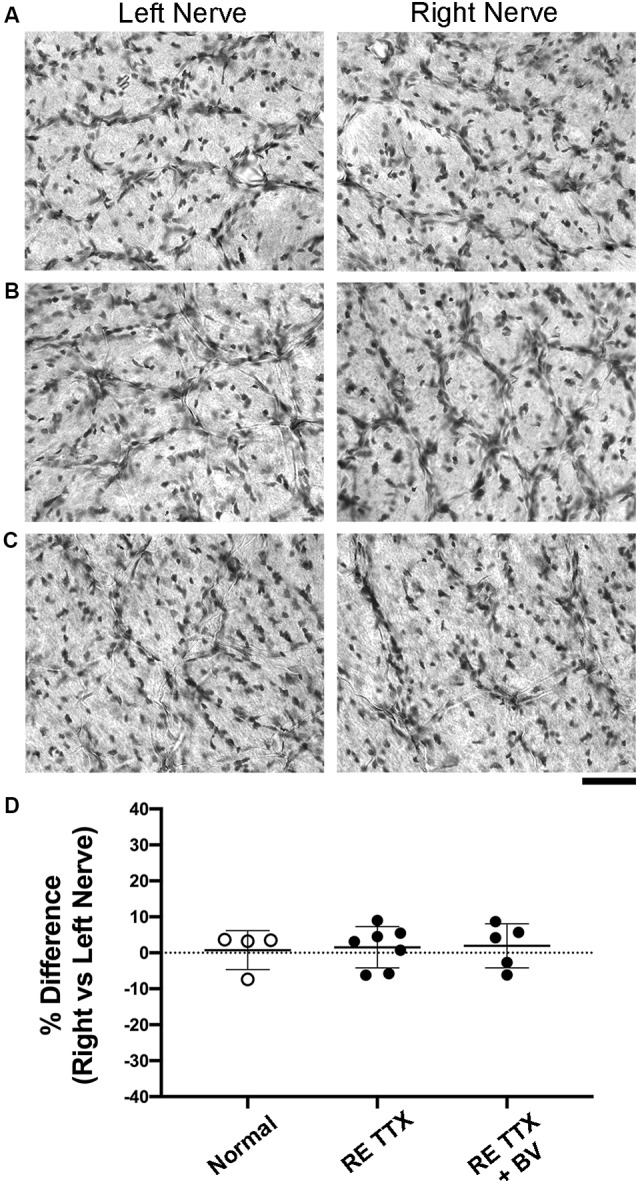
Stereological quantification of glial cell density in cross-sections of the left and right optic nerves. Glial cells were revealed with a stain for Nissl substance and in normal animals, the density and distribution of cells appeared equal across the left and right optic nerves **(A)**. Following 10 days of retinal inactivation, the left and right optics nerves maintained a normal appearance concerning the number and distributions of stained cells **(B)**, and similarity between nerves was also found after inactivation wore off **(C)**. Quantification of glial cell density **(D)** revealed an ocular dominance balance that was not different across groups (*H* = = 0.66, *p* = 0.74; Dunn’s multiple comparison test: *p* > 0.05).

**Figure 6:**
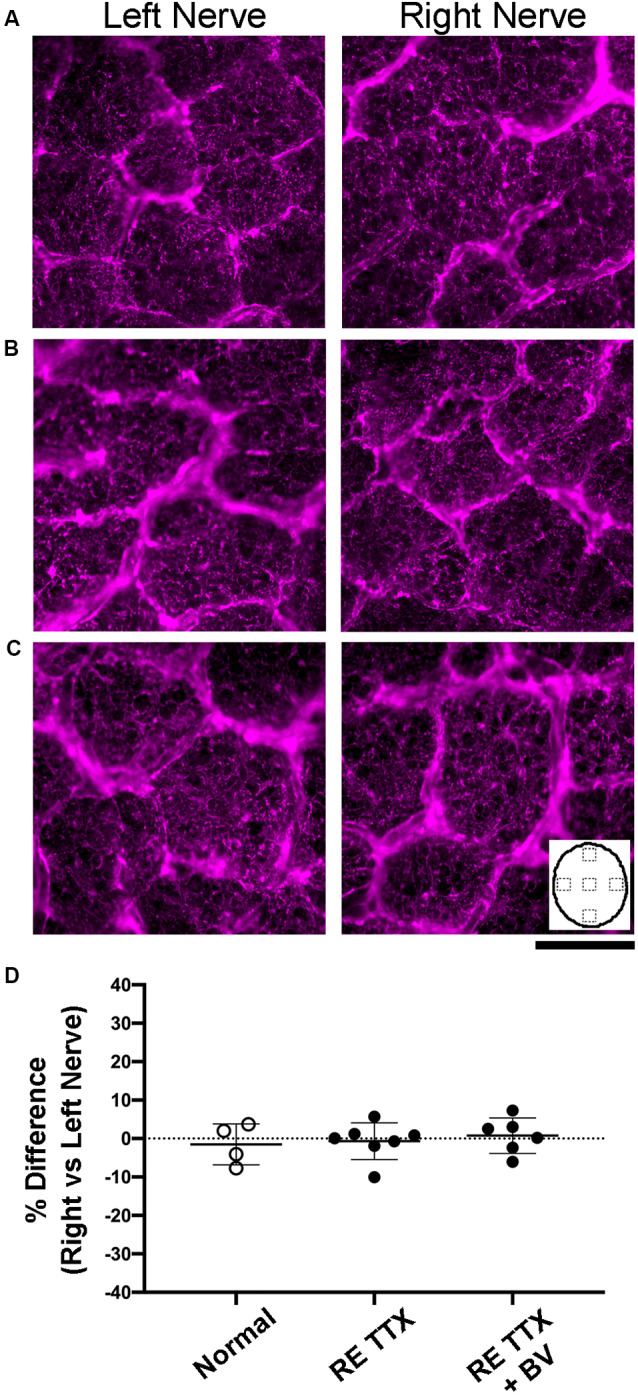
Examination of glial fibrillary acidic protein (GFAP) labeling in cross-sections of the left and right optic nerves. Immunolabeling was comparable between sections of the left and right optic nerves in normal controls **(A)**, after 10 days of right eye inactivation **(B)**, and also when right eye inactivation was followed by binocular vision **(C)**. Quantification of labeling intensity **(D)** showed a balance between the left and right nerves, as was indicated by our ocular dominance calculation, that was not different across groups (*H* = = 0.50, *p* = 0.80; Dunn’s multiple comparison test: *p* > 0.05). Inset in C depicts an outline of the optic nerve cross-section to illustrate the location of labeling measurements. Scale bar = 250 microns.

Disruption of glial cells during optic nerve degeneration plays a role in the regulation of axon loss (Son et al., 2010). Glial cell density is modified in humans, monkeys, and rodents in the context of optic nerve degeneration through a process of glial proliferation referred to as gliosis (Radius and Pederson, 1984; Jonas et al., 1995; Crish et al., 2010; Son et al., 2010). We targeted glial cells using two methods, first by staining cross-sections of the optic nerve for Nissl substance (Figure 5), and second, by using a separate set of sections to immunolabel for the glial specific intermediate filament, glial fibrillary acidic protein (GFAP; Figure 6). We observed no difference in glial Nissl staining characteristics between left and right optic nerve sections within or between the conditions we examined (Figures 5A–C). Quantification of Nissl-stained glial density was consistent with our qualitative observations, namely that densities were indistinguishable between the left and right nerves across conditions (Figure 5D). There was no significant difference in cell density between the left and right nerves within each condition (*H* = = 1.86, *p* = 0.86; Dunn’s multiple comparison test: *p* > 0.05), or between the right nerve of controls and that of the treated eye from each TTX group (Dunn’s multiple comparison test: *p* > 0.05). Immunolabeling for GFAP produced strong fluorescence in glial processes but cell somata were not easily resolved. We, therefore, measured the level of labeling fluorescence within the left and right optic nerve cross-sections. Our qualitative assessment revealed that optic nerve sections from the left and right eye were indistinguishable for each group (Figures 6A–C), and this was supported by our measurements of fluorescence that showed balanced immunolabeling between the two eyes for all three conditions (Figure 6D). Across the three groups studied, there was no statistical difference in GFAP immunofluorescence between left and right eyes [*H* = (6) = 3.71, *p* = 0.59; Dunn’s multiple comparison test: *p* > 0.05], nor was there a statistical difference between the right nerve of controls and the treated (right) nerve of TTX groups (Dunn’s multiple comparison test: *p* > 0.05).

### Visually-Evoked Potentials

**Figure 7:**
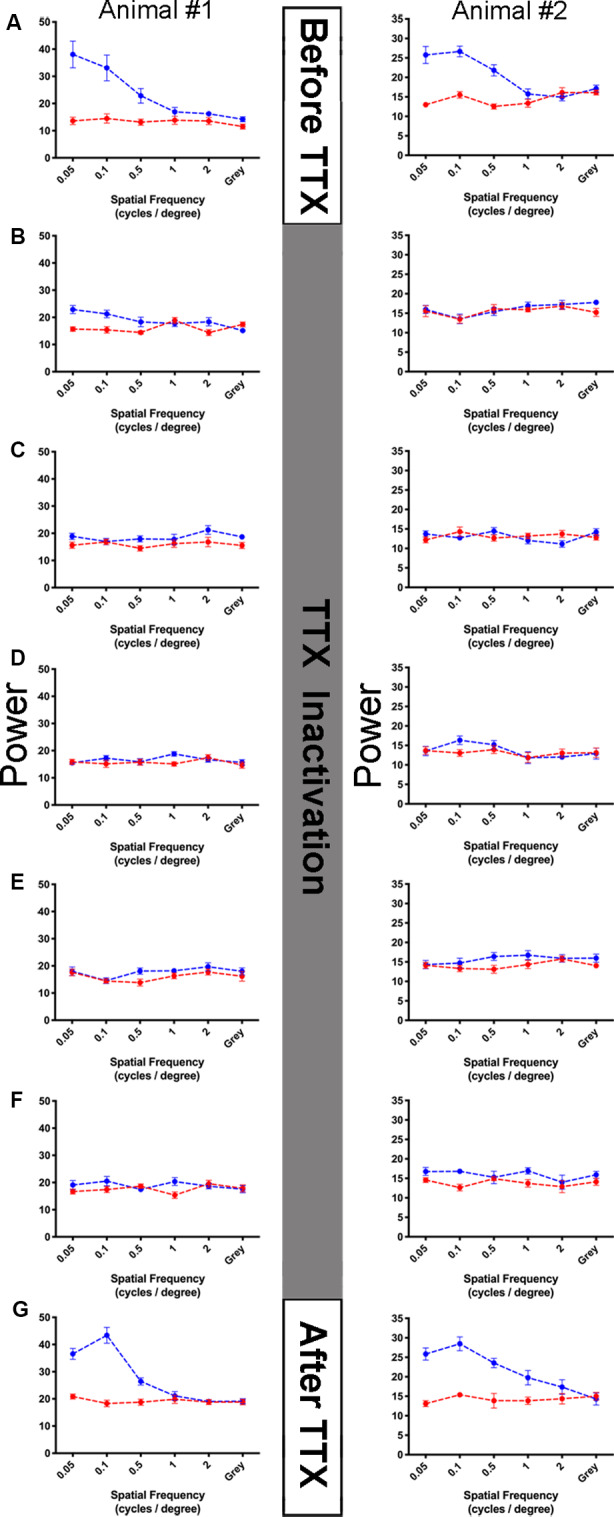
Assessment of vision function before, during, and after retinal inactivation with intraocular administration of TTX. Visually-evoked potentials were measured from the primary visual cortex of two kittens (columns) throughout the course of a TTX inactivation experiment (rows **A–G**). For each graph, spatial frequency is plotted on the abscissa, and summed power from the Fourier analysis is plotted on the ordinate (see “Materials and Methods” section). The blue points in each graph indicate the sum of visually-evoked power and red points show the non-visual baseline power (see “Materials and Methods” section). Error bars indicate standard error. No visually evoked activity should be present at the offset frequencies used to calculate the baseline, and as expected the red points form homogenously flat lines in each graph. Visually-evoked power elicited by a blank gray screen served as a control, which should be equal between the blue and red traces. Before inactivation, in both anesthetized kittens examined we found strong power at the three lowest spatial frequencies, while higher frequencies and the gray screen elicited low power that was no different than our red baseline trace **(A)**. A total of five recording sessions occurred during 10 days of inactivation, each session occurred 48 h after injection of TTX. During the period of inactivation, visual response for all stimuli was obliterated for 10 consecutive days and fell to baseline levels **(B–F)**. Ten days following the last TTX injection, the pattern of visually-evoked potentials (VEPs) for each animal was restored to that observed before inactivation was started **(G)**, indicating that our inactivation protocol did not permanently disrupt visual ability measured physiologically.

In two animals, we assessed the impact of retinal inactivation on visual function by measuring non-invasive VEPs to high contrast gratings across a range of spatial frequencies (Figure 7). Measurements of visual function taken before inactivation showed strong responses to the lower spatial frequencies presented (0.05, 0.1, 0.5 cycles/degree), and little to no cortical response beyond the baseline to the higher spatial frequencies (1, 2 cycles/degree) or a blank gray screen (Figure 7A). The pattern of visual responses was similar between the two animals we examined, and reminiscent of earlier VEP studies in anesthetized cats (Berkley and Watkins, 1971; Freeman and Marg, 1975), but lower than behavioral assessments of visual performance (Giffin and Mitchell, 1978; Mitchell, 1988). We next administered 5 intravitreal injections of TTX across 10 days and measured visual responses 48 h after each injection. In both animals, intravitreal injection of TTX obliterated VEPs so that they were equivalent to baseline levels for 10 consecutive days (Figures 7B–F). Following this inactivation period, we found that the pattern of visual responses was restored to pre-treatment levels in both animals when measurements were made 10 days after the final TTX injection (Figure 7G). That visual responses returned to pre-inactivation levels implies that retinal integrity is unaltered by brief inactivation with TTX.

## Discussion

This study examined a collection of anatomical characteristics indicative of retinal and optic nerve pathology in animals that were subjected to 10 days of monocular retinal inactivation administered as a therapy for the effects of amblyogenic rearing. We found no difference between the inactivated eye and the other eye of treated animals across all assessed characteristics, nor was there a difference when the inactivated eye was compared to normal controls. There was no evidence of a delayed histopathological response because the results observed in animals given binocular vision after inactivation treatment were not different from normal controls. Further, our measurement of retinal function using VEPs demonstrated a recovery of normal responses following 10 days of inactivation. These results are consistent with the conclusion that brief retinal inactivation does not significantly alter the anatomical integrity of the GCL or characteristics of the optic nerve. Our anatomical findings are in agreement with behavioral results in cats that show a return to normal visual acuity following 4 days of binocular retinal inactivation (Fong et al., 2016). Our results are also consistent with a human case report that revealed no evidence of retinal toxicity following intracameral administration of lidocaine, a short-lasting sodium channel blocker, injected to augment anesthesia during intraocular surgery in a human patient (Hoffman and Fine, 1997). After lidocaine injection, the patient reported complete loss of vision as indicated by the absence of light perception elicited from indirect ophthalmoscopy; however, full visual recovery was observed several hours after application of the drug implying that the inactivation did not elicit ocular pathology (Hoffman and Fine, 1997). It is important to acknowledge that our assessment of histopathology is limited to the examination of only a small number of anatomical targets relative to the scope of possibilities. Additional characteristics indicative of ocular and retinal health will need to be assessed to reach a sufficient safety threshold for retinal inactivation to be considered as a therapy for human amblyopia. For instance, confirmation that inactivation does not stimulate glial activation should include assessment of additional characteristics that are linked to this response, including ionized calcium-binding adaptor protein-1 (IBA-1; Ito et al., 1998) and examination of the structural protein vimentin (Jiang et al., 2012). Notwithstanding this critical need for further investigation, the lack of retinal and functional abnormality observed in the current study encourages the use of retinal inactivation as an effective amblyopia treatment, particularly at ages beyond the critical period when conventional treatments fail to yield significant recovery. These results also raise the possibility that retinal inactivation could be safely employed for purposes beyond the treatment of amblyopia.

We are confident that the absence of retinal abnormality revealed by the current study was not due to the ineffective administration of TTX that resulted in incomplete or absent retinal inactivation. All animals that received intraocular TTX administration exhibited functional characteristics consistent with inactivation of retinal output, including the lack of pupillary response, no visual placing behavior using the injected eye, and some of the injected animals in this investigation were subjects in a prior study that demonstrated anatomical recovery from the effects of MD after inactivation (Duffy et al., 2018). Also, visual cortical VEPs were reduced to baseline during the 10 day-inactivation period (Figure 7) indicating that our protocol for inactivation was sufficient to silence retinal output for at least 10 days.

All of the experimental animals in this study were subjected to a preceding duration of MD before retinal inactivation because they were subjects in a previous study or are part of an ongoing investigation to examine inactivation as a therapy for deprivation-induced amblyopia. The rearing history of these animals raises the possibility that the balance between the eyes that we report on all measurements could be the result of balanced pathology in the monocularly deprived (left) and inactivated (right) eyes. We believe this is unlikely for two reasons. First, MD in cats does not alter retinal characteristics such as retinal ganglion cell density, soma size, or function (Cleland et al., 1980; Spear and Hou, 1990). Even when MD is imposed for up to 7 years, retinal ganglion cell density and soma area measured from flat-mounted sections of retina were not different between deprived and non-deprived eyes within area centralis, peripheral binocular segment, or monocular segment (Spear and Hou, 1990). Second, when compared to normal control eyes, all of the measurements obtained from the inactivated eyes of experimental animals were not different, indicating that the anatomical characteristics that we examined in this study following retinal inactivation were not different from normal.

Results from our study indicate that inactivation of retinal cells at the ages examined does not lead to cell death or neural degeneration. At much younger ages in rodents, retinal inactivation with TTX can alter the extent of natural cell death in the retina. Inactivation of one or both eyes in 2-week-old rats leads to an increase in the survival of retinal ganglion cells, which is believed to result from a decrease in the activity-dependent competition of eye inputs to the superior colliculus (Fawcett et al., 1984). In tree shrews, inactivation of both eyes starting at birth and lasting 2 weeks does not alter the normal segregation of eye inputs to layers of the lateral geniculate nucleus; however, this early inactivation slows the normal pace of LGN development (Casagrande and Condo, 1988). Consistent with results from the current study, inactivation of one eye for 3 weeks starting at postnatal day 9 in cats does not alter retinal cell density; however, the regular mosaic of ON and OFF α ganglion cell pairs was decreased suggesting that the normal arrangement of cell types is altered by long durations of retinal inactivation when started early in life (Jeyarasasingam et al., 1998). Studies in which animals were subjected to retinal inactivation at older ages beyond early development report no abnormalities in the inactivated eye. Intraocular administration of TTX in adult cats for 1–6 weeks produced no detectable evidence of trauma in the injected eye, with eyes maintaining clear vitreous and near-invisible injection sites, and the retina of injected eyes appeared unremarkable upon microscopic inspection with no evidence of morphological deterioration (Wong-Riley and Riley, 1983). In post-critical period macaque monkeys, monocular inactivation lasting 4 weeks produced a mild decrease in the metabolic marker, cytochrome oxidase, within large ganglion cells as well as those in the outer and inner plexiform layers, but apart from this mild change, retinae were histologically intact and not different from controls (Wong-Riley and Carroll, 1984). In cats, similar cytochrome oxidase reductions following 1–6 weeks of retinal inactivation are observed within the inactivated-eye layers of the lateral geniculate nucleus and in the visual cortex, but cytochrome levels were restored to normal with the provision of binocular vision and the wearing off of inactivation (Wong-Riley and Riley, 1983). In aggregate, these results indicate that in some species retinal inactivation introduced shortly after birth can alter the normal development of the retina by at least promoting cell survival and altering cell mosaics in the GCL; however, outside of early life, it appears the retina is impervious to major modification consequent to even extended durations of inactivation produced by TTX. These conclusions are buttressed by our physiological findings that VEPs in cats are restored to normal when inactivation of the retina wears off (Figure 7).

Conventional therapies for human amblyopia such as patching, atropine and Bangerter filter, have been employed for centuries to treat amblyopia (Hoyt, 2015) but are limited in their ability to produce recovery beyond about the age of 7 years, and are impeded by poor compliance that can be magnified by the requirement for long treatment duration (Stewart et al., 2003; Holmes et al., 2011; Holmes and Levi, 2018). Stimulus deprivation-induced amblyopia is a particularly severe form of the disorder and can be unresponsive to mainstay therapy ultimately resulting in poor visual outcomes even with adequate treatment conditions (Hatt et al., 2006). In its current form, occlusion therapy aims to maximize recovery of the amblyopic eye without adversely affecting the fellow eye, which serves to provide a good second eye in case the better-seeing eye is lost or damaged (Rahi et al., 2002; Hatt et al., 2006). The introduction of a novel penalization therapy that enhances and expedites recovery outcomes over conventional treatments could offer a significant advancement in the remediation of amblyopia. That abnormalities provoked by MD can be restored to normal following brief inactivation of the fellow eye (Duffy et al., 2018) or both eyes (Fong et al., 2016) raises the intriguing possibility that either therapy could be used to stimulate recovery, and it appears this can occur without ocular detriment.

## Data Availability Statement

All datasets presented in this study are included in the article/Supplementary Material.

## Ethics Statement

All procedures in this study were approved by the standing committee overseeing animal care and ethics at Dalhousie University, and adhered to use guidelines detailed by the Canadian Council on Animal Care.

## Author Contributions

KD designed the research. KD and ND performed the anatomy. KD, BK, and NC performed the physiology. KD composed the figures and wrote the article.

## Conflict of Interest

The authors declare that the research was conducted in the absence of any commercial or financial relationships that could be construed as a potential conflict of interest.
