# Supplementary material for: Retinal and Optic Nerve Integrity Following Monocular Inactivation for the Treatment of Amblyopia
Source: Front Syst Neurosci. 2020 Jun 10;14:32. doi: 10.3389/fnsys.2020.00032 (PMC7298113; doi:10.3389/fnsys.2020.00032)
Supplement: Supplementary file 1 [file Table_1.docx]

| Animal | Condition | Rearing | GCL Density  (cells / mm^2^)  LE RE | | GCL Soma Size (μm^2^)  LE RE | |
| --- | --- | --- | --- | --- | --- | --- |
| 409 | Normal | 10 wk | 754 | 737 | 54 | 54 |
|  |  |  | 2.25% | | 0% | |
| 453 | Normal | 14 wk +1day buffer RE | 741 | 730 | 59 | 57 |
|  |  |  | 1.48% | | 3.38% | |
| 443 | RE TTX | 6 wk MD + 10 day TTX + 20 day BV | 751 | 762 | 62 | 55 |
|  |  |  | -1.46% | | 11.2% | |
| 444 | RE TTX | 6 wk MD + 10 day TTX + 20 day BV | 742 | 796 | 57 | 58 |
|  |  |  | -7.27% | | -1.75% | |
| 450 | RE TTX | 10 wk MD + 10 day TTX + 20 day BV | 786 | 796 | 61 | 59 |
|  |  |  | -1.27% | | 3.27% | |
| 452 | RE TTX | 10 wk MD + 10 day TTX + 20 day BV | 753 | 758 | 52 | 57 |
|  |  |  | -0.66% | | -9.61% | |

*Table 1: Measurements of cell density and soma size within the GCL. MD was performed on postnatal day 30 by lid suture of the left eye (LE). All TTX injections were made into the right eye (RE TTX). For each cell: left eye (LE) measurements on left, right eye (RE) measurements on right. Presented measurements are the averages across 3 sagittal sections for each retina. For some animals binocular vision (BV) was provided after inactivation wore off. Percentages indicate: % Difference (ODI) = ((LE-RE)/LE) x 100.*
