# Supplementary material for: Retinal and Optic Nerve Integrity Following Monocular Inactivation for the Treatment of Amblyopia
Source: Front Syst Neurosci. 2020 Jun 10;14:32. doi: 10.3389/fnsys.2020.00032 (PMC7298113; doi:10.3389/fnsys.2020.00032)
Supplement: Supplementary file 2 [file Table_2.docx]

| Animal | Condition | Rearing | NF Intensity  LE RE | | Luxol Intensity  LE RE | | GFAP Intensity  LE RE | | Glial Cell Density  (cells / mm^2)^  LE RE | |
| --- | --- | --- | --- | --- | --- | --- | --- | --- | --- | --- |
| 410* | Normal | 14wk +10day buffer RE | 49.38 | 51.02 | 113.05 | 119.62 | 90.92 | 94.62 | 8497 | 9125 |
|  |  |  | -3.32% | | -5.81% | | -4.08% | | -7.39% | |
| 453* | Normal | 14wk +1day buffer RE | 107.91 | 106.81 | 149.43 | 145.55 | 106.68 | 102.70 | 8612 | 8298 |
|  |  |  | 1.02% | | 2.59% | | 3.73% | | 3.65% | |
| 466 | Normal | 40day | 70.95 | 75.34 | 112.78 | 122.44 | 122.34 | 131.80 | 6370 | 6166 |
|  |  |  | -6.19% | | -8.56% | | -7.73% | | 3.20% | |
| 468 | Normal | 40day | 68.13 | 72.01 | 149.95 | 154.12 | 119.88 | 117.46 | 8304 | 8012 |
|  |  |  | -5.68% | | -2.77% | | 2.02% | | 3.52% | |
| 442 | RE TTX | 6wk MD + 10day TTX | 72.02 | 73.03 | 138.48 | 141.60 | 117.55 | 117.46 | 7841 | 7411 |
|  |  |  | -1.40% | | -2.25% | | 0.07% | | 5.48% | |
| 404 | RE TTX | 10wk MD + 10day TTX | 117.58 | 116.72 | 126.17 | 135.76 | 135.80 | 136.79 | 7224 | 6998. |
|  |  |  | 0.73% | | -7.60% | | -0.73% | | 3.13% | |
| 451 | RE TTX | 10wk MD + 10day TTX | 77.06 | 79.87 | 153.49 | 149.91 | 113.18 | 106.75 | 7802 | 7102 |
|  |  |  | -3.65% | | 2.33% | | 5.68% | | 8.97% | |
| 377 | RE TTX | 14wk MD +10day TTX | 102.80 | 104.97 | 152.11 | 159.07 | 99.45 | 109.42 | 7885 | 7830 |
|  |  |  | -2.12% | | -4.58% | | -10.03% | | 0.70% | |
| 369 | RETTX | 16wk MD +10day TTX | 115.89 | 118.33 | 149.19 | 152.45 | 123.31 | 121.80 | 8833 | 8436 |
|  |  |  | -2.11% | | -2.19% | | 1.22% | | 4.49% | |
| 368 | RETTX | 18wk MD + 10day TTX | 79.93 | 80.91 | 129.37 | 117.97 | 106.74 | 105.84 | 7720 | 8199 |
|  |  |  | -1.23% | | 8.82% | | 0.84% | | -6.20% | |
| 381 | RE TTX | 6wk MD +10day TTX | NA | | 173.10 | 181.84 | 103.57 | 105.53 | 6843 | 7240 |
|  |  |  |  |  | -5.05% | | -1.89% | | -5.80% | |
| 441 | RE TTX+BV | 6wk MD + 10day TTX + 20day BV | 70.46 | 74.92 | 158.42 | 156.41 | 85.74 | 90.95 | 8521 | 8753 |
|  |  |  | -6.34% | | 1.27% | | -6.07% | | -2.72% | |
| 412 | RE TTX+BV | 10wk MD + 10day TTX +20day BV | 68.38 | 70.19 | 155.60 | 159.83 | 110.74 | 110.50 | 7367 | 6948 |
|  |  |  | -2.66% | | -2.72% | | 0.21% | | 5.68% | |
| 413 | RE TTX+BV | 10wk MD + 10day TTX + 10day BV | 81.13 | 86.66 | 164.70 | 157.75 | 112.54 | 104.38 | 8282 | 7934 |
|  |  |  | -6.82% | | 4.22% | | 7.25% | | 4.19% | |
| 450 | RE TTX+BV | 10wk MD + 10day TTX + 20day BV | 58.57 | 59.34 | 131.33 | 129.75 | 111.94 | 109.11 | 6645 | 7053 |
|  |  |  | -1.31% | | 1.20% | | 2.52% | | -6.14% | |
| 452 | RE TTX+BV | 10wk MD +10day TTX +20day BV | 75.78 | 76.26 | 137.54 | 151.05 | 110.89 | 107.57 | 8596 | 7852 |
|  |  |  | -0.64% | | -9.82% | | 2.99% | | 8.65% | |
| 456 | RE TTX+BV | 15wk MD +10day TTX + 12day BV | 103.67 | 99.70 | 179.13 | 175.84 | 96.21 | 98.52 | NA | |
|  |  |  | 3.82% | | 1.83% | | -2.39% | |  |  |

*Table 2: Measurements of neurofilament labeling, myelin (luxol) staining, GFAP labeling, and Glial cell density in cross-sections of the optic nerve. MD was performed on postnatal day 30 by lid suture of the left eye (LE). All TTX injections were made into the right eye (RE TTX). For each cell: left eye (LE) measurements on left, right eye (RE) measurements on right. For some animals binocular vision (BV) was provided after inactivation wore off. Percentages indicate: % Difference (ODI) = ((LE-RE)/LE) x 100. Intensity measurements are averaged mean grey values obtained from five image samples taken from each optic nerve. NA indicates that reaction product was poor and not suitable for measurement. Asterisk indicates controls that received monocular vehicle injections.*
